# Impact of (nano ZnO/multi-wall CNTs) prepared by arc discharge method on the removal efficiency of stable iodine ^127^I and radioactive iodine ^131^I from water

**DOI:** 10.1038/s41598-024-54604-w

**Published:** 2024-02-20

**Authors:** Ahmed M. El-Khatib, I. I. Bondouk, Kh. M. Omar, Ahmed Hamdy, Mahmoud I. Abbas, M. El-Khatib, Sabbah I. Hammoury, Mona M. Gouda

**Affiliations:** 1 Physics Department, Faculty of Science, Alexandria University, Alexandria, Egypt; 2 Physics Department, Faculty of Science, Tanta University, Tanta, Egypt; 3 Obtained Philosophy Doctoral Degree in Nuclear Physics, Physics Department, Faculty of Science, Tanta University, Tanta, Egypt; 4 Basic Sciences Department, Faculty of Engineering, Pharos University, Alexandria, Egypt; 5 Head of Medical Physics and Radiotherapy Department, Alexandria Ayadi Almostakbal Oncology Hospital, Alexandria, Egypt

**Keywords:** Nano ZnO*/*MWCNTs, Nano adsorbents, Iodine 127, Iodine 131, Water treatments, Kinetic models, Isotherm models, Environmental sciences, Nanoscience and technology, Physics

## Abstract

Radioactive iodine isotopes especially ^131^I are used for diagnosis and treatment of different types of cancer diseases. Due to the leak of radioactive iodine into the patient’s urine in turn, the wastewater would be contaminated, so it is worth preparing a novel adsorption green material to remove the radioactive iodine from wastewater efficiently. The removal of ^127^I and ^131^I contaminants from aqueous solution is a problem of interest. Therefore, this work presents a new study for removing the stable iodine ^127^I^−^ and radioactive iodine ^131^I from aqueous solutions by using the novel nano adsorbent (Nano ZnO/MWCNTs) which is synthesized by the arc discharge method. It is an economic method for treating contaminated water from undesired dissolved iodine isotopes. The optimal conditions for maximum removal are (5 mg/100 ml) as optimum dose with shacking (200 rpm) for contact time of (60 min), at (25 °C) in an acidic medium of (pH = 5). After the adsorption process, the solution is filtrated and the residual iodide (^127^I^−^) is measured at a maximum UV wavelength absorbance of 225 nm. The maximum adsorption capacity is (15.25 mg/g); therefore the prepared nano adsorbent (Nano ZnO/MWCNTs) is suitable for treating polluted water from low iodide concentrations. The adsorption mechanism of ^127^I^−^ on to the surface of (Nano ZnO/MWCNTs) is multilayer physical adsorption according to Freundlich isotherm model and obeys the Pseudo-first order kinetic model. According to Temkin isotherm model the adsorption is exothermic. The removal efficiency of Nano ZnO/MWCNTs for stable iodine (^127^I^−^) from aqueous solutions has reached 97.23%, 89.75%, and 64.78% in case of initial concentrations; 0.1843 ppm, 0.5014 ppm and 1.0331 ppm, respectively. For the prepared radio iodine (^131^I^−^) solution of radioactivity (20 µCi), the dose of nano adsorbent was (10 mg/100 ml) and the contact time was (60 min) at (pH = 5) with shacking (200 rpm) at (25 °C). The filtration process was done by using a syringe filter of a pore size (450 nm) after 2 days to equilibrate. The removal efficiency reached (34.16%) after the first cycle of treatment and the percentage of residual radio iodine was (65.86%). The removal efficiency reached (94.76%) after five cycles of treatment and the percentage of residual radio iodine was (5.24%). This last percentage was less than (42.15%) which produces due to the natural decay during 10 days.

## Introduction

Living organisms can’t produce iodine and the iodide (I^−^) is delivered with food and drinking water. The iodide anion (I^−^) is as an electron donor and it is oxidized in the process of thyroid hormone synthesis by Thyroperoxidase. The (Na^+^/I^−^ symporter, NIS) is the responsible for the active transport of iodine to the thyroid and thyroperoxidase. The risk of papillary thyroid cancer (TPO) is increased with the increased exposure to iodine^1^. Iodine salts are dissolved in the water of oceans, seas, rivers, and lakes. In seawater, the average concentration of iodine is in the range of (45–60 µg/L) while it is in the range of (0.5–20 µg/L) for river and lake water. The taste and odour thresholds for iodine in water are between (0.147 and 0.204 mg/L). In the USA, the mean concentration of total iodine in drinking water is (4 μg/L) with a maximum concentration of (18 μg/L)^2^.

Iodine is used in different fields; “Povidone Iodine” is an antibacterial reagent against Gram-positive and negative organisms^3^. The different iodine compounds are used as catalysts in chemical industries while the industrial wastes pollute the environment; air, oceans, seas, rivers, and lakes. For example: **1-**Acetic acid is produced by carbonylation of methanol by using hydrogen iodine catalyst. **2-**Nylon fibers and polyamide plastics are stabilized by using copper iodine as catalyst^4^. The radioactive iodine isotopes; (^124^I, ^125^I, and ^131^I) are used in nuclear medicine^4^. ^131^I isotope plays an important role in diagnose and treat various thyroid diseases, moreover it is used for diagnostic and therapeutic purposes of Neuroblastomas, Pheochromocytomas and Paragangliomas^5,6^. ^131^I-Hippuran, ^131^I-MIBG, Oral-NaI^131^and ^131^I-capsules are prepared for use in the purposes of nuclear medicine^4,5^. It is noticed that residual traces of radioactive iodine isotopes are present in the urine of patients receiving the radioactive iodine therapy. Therefore, wastewater may be contaminated with radioactive iodine isotopes^7^.

**Figure 1:**
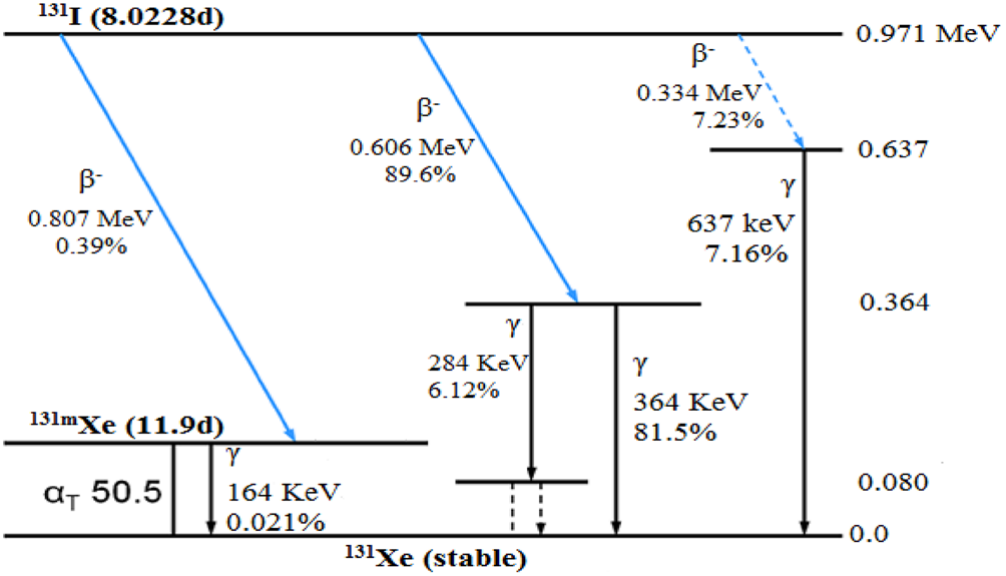
Decay Scheme of Radioactive Iodine (^131^I)^9^**.**

By irradiating the natural Tellurium dioxide (^130^TeO_2_) targets with neutrons, the radioactive tellurium (^131^Te) is produced and decays via β^−^ emission to the radioactive iodine (^131^I)^6–8^. The half-life time of (^131^I) is about 8.023 days as indicated in Fig. 1. For radioactive iodine ^131^I accidents and nuclear tests, it takes about 3 months to decay to ^131^Xe. It is concentrated in salivary glands and thyroid gastric mucosa. Urinary excretion is a predomination route (35–75% in 24 h) although there is the same fecal extraction as well. A high incidence of thyroid cancer was reported in heavily contaminated areas of the Chornobyl accident (Table 1)^10^.

**Table 1:** Summary of radioactive-iodine (^131^I) Decay ^9^.

Radioactive iodine	decay mode	Decay nuclear reaction
Half life time of ^131^I is 8.023 days	β^−^ (89.6%)	^131^I → ^131^Xe* + e^-^ + $\overline{v}_{e}$ + 0.606 MeV ^131^Xe* → ^131^Xe + γ + 0.364 MeV (81.5%) ^131^Xe* → ^131^Xe + γ + 0.284 MeV (6.12%)
	β^−^ (7.23%)	^131^I → ^131^Xe* + e^-^ + $\overline{v}_{e}$ + 0.334 MeV ^131^Xe* → ^131^Xe + γ + 0.637 MeV (7.16%)

The physical and chemical properties of Nanomaterials are different from the micro-scale sizes of the same material**.** There are more atoms on their surfaces**,** and this leads to an increase in the surface area of Nano-material relative to the volume. Therefore**,** Nano-materials are considered as effective chemical adsorbents and catalysts because more chemicals can interact with them simultaneously^11^. High heat and electrical conductivity, structural feature and exceptional sorption capacities of carbon nano tubes (CNTs) make them an important nano sorbent^12^. Nanocomposites which contain (CNTs) have high capacities to remove dissolved heavy metals and dyes from contaminated water^13^. Several studies have focused on how to remove stable and radioactive active iodine isotopes from water by using different sorbents and methods. A lot of these methods were not economical due to use various expensive nano adsorbents, for examples; (MXene/AgNW) composite material iodine from water^14^, (Mn_3_O_4_@polyaniline nanocomposite with multiple active sites)^15^, (AgNPs/CAM)^16^, (Ag@Cu-based Metal–organic framework)^17^, (AgNPs-Ag_2_O NPs modified Al_2_O_3_)^18^, (AgNPs-impregnated zeolites)^19^, (Ag/Fe_3_O_4_ composite nano-adsorbent)^20^, (Core–shell ZnO/Cu_2_O encapsulated Ag nanoparticles nano-composites)^21^, (Silver-Impregnated Magnetite Mesoporous Silica Composites)^22^ and (Diatomite-nano TiO_2_ composite)^23^.

In previous work, the removal of two radioactive isotopes (^65^Zn and ^60^Co) from aqueous solutions has been studied by using different nanocomposites like; (TiO_2_/Ag_2_O Nanocomposites)^24^, (Polyaniline-silver oxide)^25^ and (Sodium Nano Bentonite coated with Oleyl-amine)^26^_**.**_ The values of removal efficiency for ^65^Zn and ^60^Co were between (90% and 94%)^24–26^. The study in this work, presents new method for the removing of dissolved stable iodide (^127^I^−^) and radioactive iodide (^131^I^−^) from the prepared aqueous solutions using novel nan adsorbent (Nano ZnO/MWCNTs) prepared by the arc discharge^27^.

## Chemicals and instruments

### Chemicals

In a previous work^27^ the nanocomposite (Nano ZnO/MWCNTs) has been prepared by the arc discharge method by using an alternating electrical current (15A) at a constant voltage (70 V). The (Nano ZnO/MWCNTs) has been used as a nano adsorbent in the presented work. Radioactive iodine (^131^I) was supplied by Ayady Hospital, Alexandria, Egypt. Stable iodine (^127^I) solution (5%) has been diluted to solutions with different concentrations. The pH values of diluted iodine solutions have been adjusted (from 5 to 14) by using Glacial acetic acid and sodium hydroxide solution NaOH (1.0 N).

### Instruments

**Figure 2:**
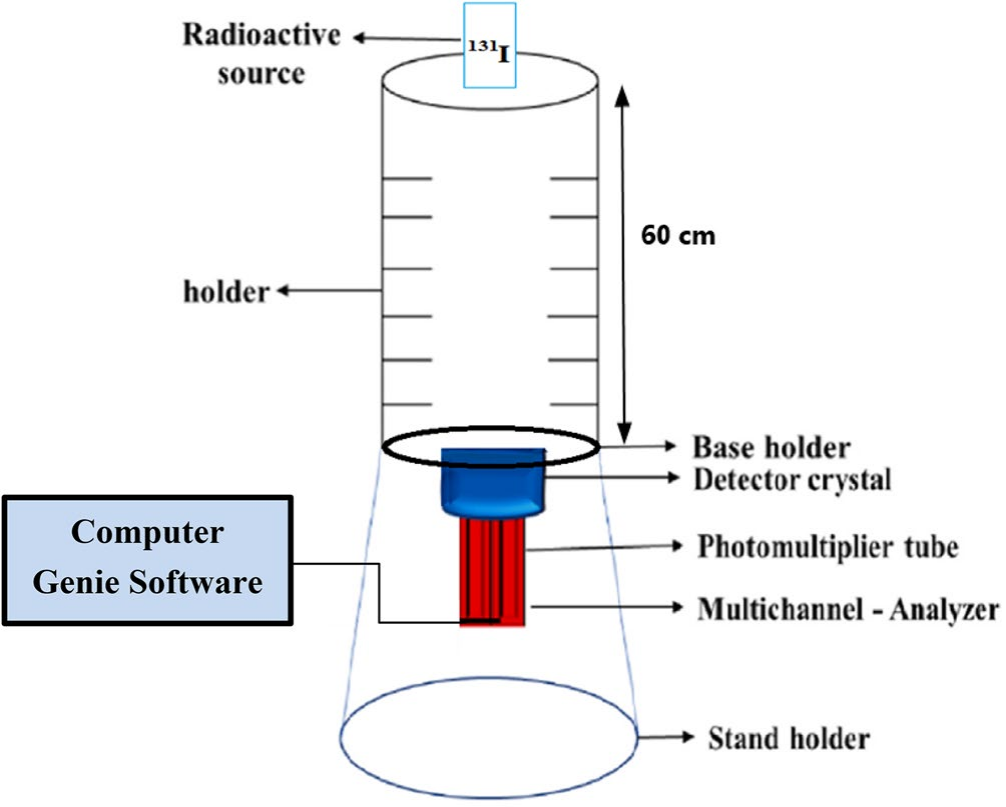
Experimental setup configuration Schematic diagram of 3X3 NaI(Tl) scintillation detector.

Shaking Water Bath; (JULABO, D-77960 Seelbach/Germany) has been used in this work. All measurements for stable iodine (^127^I^−^) have been done by using Thermo Spectronic Device (Hellos Alpha, 9423 1002E) at a maximum UV wavelength absorbance of (225 nm)^14,20^. The measurements of radioactive iodine (^131^I^−^) in aqueous solutions before and after applying the nano adsorbent have been measured by using a 3X3 NaI (Tl) scintillation detector ^28–31^. In order for the beam of photons to be narrow, it is necessary for the source-detector distance to be ten times the diameter of the detector^28^. Consequently, the source is positioned at an axial distance of 60 cm from the detector cap. The spectral analysis was carried out using Genie 2000 Software program. The schematic diagram of 3X3 NaI(Tl) scintillation detector is illustrated in the Fig. 2. The study of removing the dissolved radioactive iodine (^131^I^−^) from the prepared aqueous solution by using the nano adsorbent (Nano ZnO/MWCNTs) has been carried out in the Radiation Physics Laboratory, Faculty of Science, Alexandria University, Egypt.

## Experimental method

The stable iodine (^127^I) has the same chemical properties as the radioactive iodine (^131^I)^23^. Therefore, ^127^I had been used in order to determine the best conditions of adsorption before the adsorption of ^131^I was studied. To identify the optimal conditions for high (^127^I^−^) removal performance by using the nano adsorbent (Nano ZnO/MWCNTs); the following experiments were carried out in the Central Laboratory of the Faculty of Pharmacy, Alexandria University, Egypt. Several experimental steps were carried out to find the best contact time (T_Best_), the optimum nano adsorbent dose (D_opt_) at (T_Best_), and the suitable pH value at both (D_opt_) and (T_Best_). In order to find these parameters one has to change one parameter and determine the removal efficiency percentage (%) while keeping the others constant. In these experiments, the nano adsorbent (Nano ZnO/MWCNTs) had been settling before the residual concentration of iodide (^127^I^−^) in the supernatant solution was measured spectrophotometrically maximum UV wavelength absorbance of 225 nm^14,20^

To find (T_Best_), the contact time was varied {15 min, 30 min, 45 min, 60 min, 75 min, 90 min} at a constant initial concentration (0.1843 ppm), nano adsorbent dose (4 mg/100 ml), temperature (25 °C) and (pH = 7) with shacking (200 rpm) in a shaking water bath. Similarly, the nano adsorbent dose at (T_Best_) was varied from 1.0 mg/100 ml to 10.0 mg/100 ml at constant initial concentration (0.1843 ppm), contact time (T_Best_), temperature (25 °C) and (pH = 7) with shacking at 200 rpm to find the optimum nano adsorbent dose (D_opt_) at the best contact time (T_Best_). The pH value was varied from 4 to 14 to determine the suitable pH value for the adsorption process at constant initial concentration, nano adsorbent dose (D_opt_), contact time (T_Best_), and temperature (25 °C) with shacking at 200 rpm. Referring to the two Eqs. (1) and (2), the removal efficiency (%) and the adsorption capacity *q*_t_ (mg/g) of nano adsorbent (Nano ZnO/MWCNTs) for the dissolved iodine (^127^I) were calculated^14,32,33^.

1 $$ {\text{Remval Efficiency }}\left( {{\% }} \right) = \frac{{\left( {{\text{C}}_{{\text{o}}} - {\text{C}}_{{\text{t}}} } \right)}}{{{\text{C}}_{{\text{o}}} }} \times 100 $$

2 $$ {\text{q}}_{{\text{t}}} = \frac{{\left( {{\text{C}}_{{\text{o}}} - {\text{C}}_{{\text{t}}} } \right) \times {\text{V}}}}{m} $$

where C_0_ is the initial concentration of iodine (^127^I^−^) dissolved in deionized water, C_t_ is the residual concentration of iodine (^127^I^−^) after a given contact time (t),V is the volume of solution (100 ml) and m is the mass of the nano adsorbent dose (gram)**.**

## Results and discussion

### Impact of contact time

**Figure 3:**
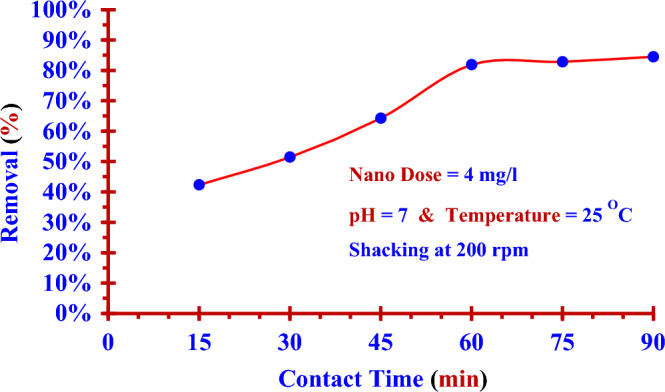
Removal efficiencies of (Nano ZnO/MWCNTs) for (^127^I^−^) from aqueous solutions versus contact times. (± 0.0044).

The dose (4.0 mg) of nano adsorbent (Nano ZnO/MWCNTs) was added to (100 ml) of the iodine (^127^I^−^) solution with a concentration of (0.1843 ppm). The contact time was varied {15, 30,…,90 min} at (pH = 7.0) and (25 °C) with shaking (200 rpm). Figure 3 shows the equilibrium contact time was attained within 60 min which is considered to be the best contact time for all investigated samples. The removal efficiency was (81.88%) for iodine (^127^I). It takes 30 min more to increase the removal efficiency by (2.6%) to reach (84.48%).

### Impact of nano adsorbent dosage

Different doses of Nano ZnO/MWCNTs (1.0–10.0 mg) were added to 100 ml of the iodine (^127^I^−^) solution (0.1843 ppm) and shacked for (60 min) with shaking (200 rpm) at (25 °C) and (pH = 7).

**Figure 4:**
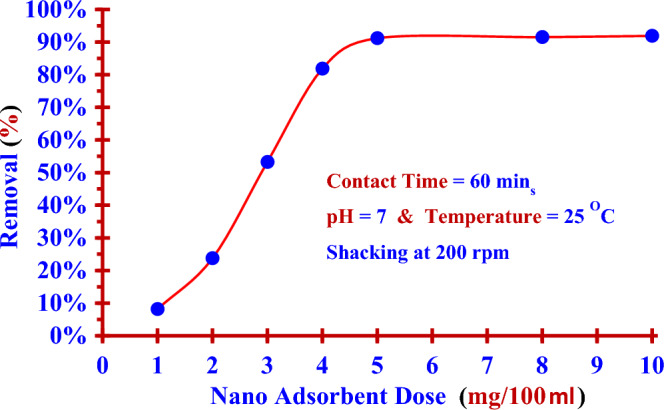
Removal efficiencies for (^127^I^−^) from aqueous solutions versus doses of (Nano ZnO/MWCNTs).

Figure 4 illustrates the impact of nano adsorbent dose on the removal efficiency of iodide (^127^I^−^). The results clearly that the dose of nano adsorbent (5 mg/100 ml) was the optimum dose to give removal efficiency (91.21%). As the nano adsorbent dose increased (from 1 to 5 mg/100 ml), there was a remarkable increase in the removal efficiency. This increase in removal efficiency slowed down to show saturation for the extra add nano adsorbent dose up to (10 mg/100 ml) with removal efficiency (91.92%).

### Effect of pH on the adsorption process

The dose (5.0 mg) of (Nano ZnO/MWCNTs) was added to (100 ml) of the iodine (^127^I) solution (0.5014 ppm) and shacked for (60 min) with shaking (200 rpm) at (25 °C) while pH value of the solution was varied from 4 to12.

**Figure 5:**
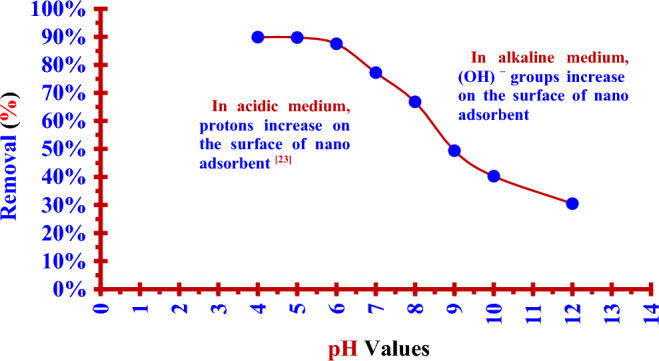
Chart between the pH of iodide (^127^I^−^) solution and the removal efficiency of nano adsorbent (Nano ZnO/MWCNTs).

**Figure 6:**
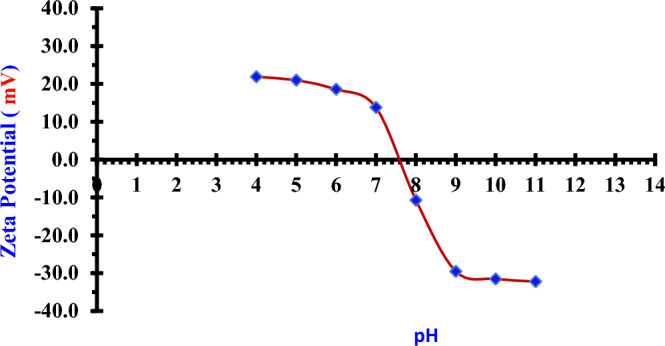
Zeta potential of the (Nano ZnO/ MWCNTs) in deionized water at different pH values.

Figure 5 depicts these results to get the saturation removal % in an acidic medium at (pH = 5) for all investigated solutions. The removal efficiency of (Nano ZnO/MWCNTs) for iodide (^127^I^−^) from an aqueous solution (0.5014 ppm) increases with decreasing pH. The removal efficiency was (89.75%) and (89.89%) at (pH = 5) and (pH = 4), respectively. The increase in removal efficiency slowed down at (pH < 5). Therefore the saturation was achieved at (pH = 5) which is suitable for the study of adsorption process. The Fig. 6 displays the value of Zeta potentials for the prepared nano composite (Nano ZnO/ MWCNTs) that prepared by arc discharge method at (15 A) in deionized water at different pH values. The Pezo Electric point is clear at (pH = 7.6). At pH values lower than point of zero charge (pHpzc = 7.6), the surface of (Nano ZnO/MWCNTs) is protonated due to the increase of protons^25^. So, the surface of MWCNTs will be more positive, and hence it will be suitable for adsorbing the iodinde (^127^I^−^) in the acidic medium.

### Impact of initial concentration (C_0_)

**Figure 7:**
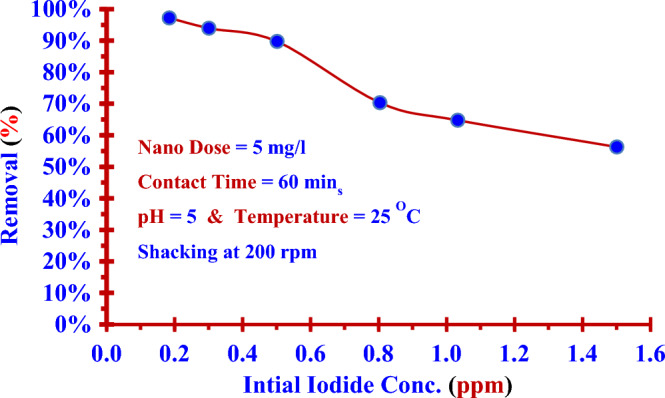
Chart between the initial concentrations of stable iodide ^127^I^−^ versus the removal efficiencies of (Nano ZnO/MWCNTs) at the optimum conditions. (± 0.0047).

The (Nano ZnO/MWCNTs) was used at the optimum conditions to remove iodide (^127^I^−^) from prepared solutions with different initial concentrations at the optimum conditions. The chart in Fig. 7 indicates the removal efficiency of (Nano ZnO/MWCNTs) for iodide (^127^I^−^) from aqueous solutions decreases with the increasing of its initial concentration.

## Kinetics Aspects; pseudo-first and second-order kinetic models

The initial concentration for iodide (^127^I^−^) dissolved in deionized water is (C_0_). The residual concentration is (C_t_) after a contact time (t) with the optimum dose of nano adsorbent (Nano ZnO/MWCNTs). In this work, the adsorption kinetic of nano adsorbent for iodide (^127^I^−^) from aqueous solutions has been described by the pseudo-first and second-order kinetic models under optimum conditions^14,32,33^.

**Figure 8:**
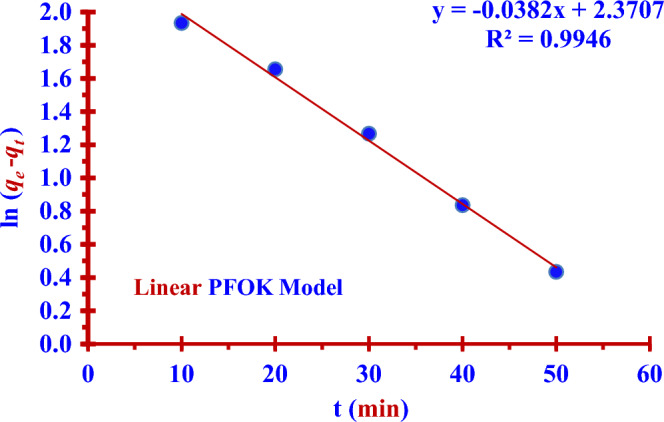
Linear PFOK model for (^127^I^−^) adsorption.

**Figure 9:**
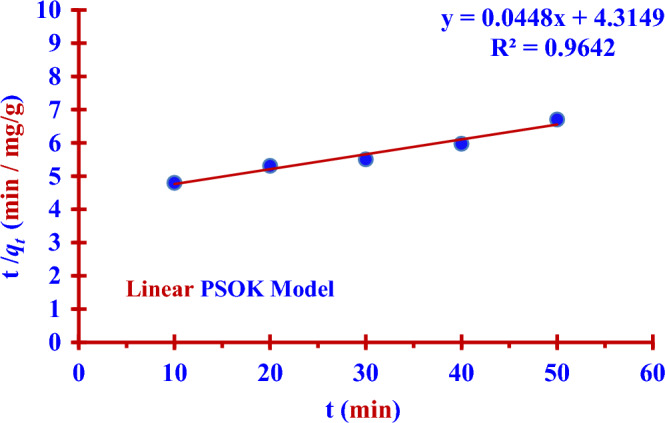
Linear PSOK model for (^127^I^−^) adsorption.

**Figure 10:**
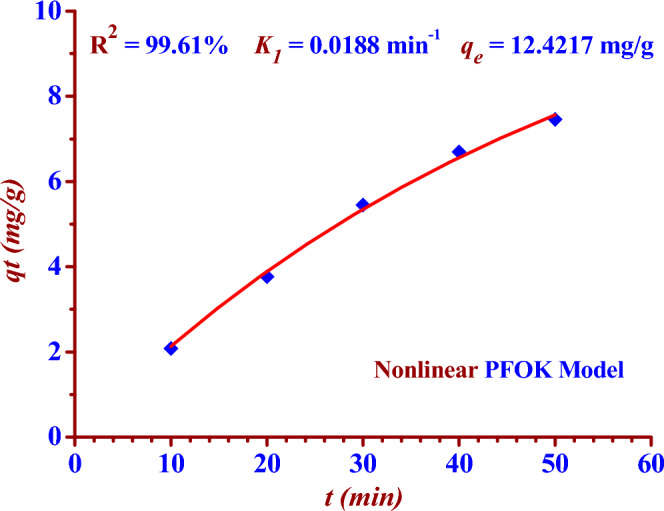
Nonlinear PFOK model for (^127^I^−^) adsorption.

**Figure 11:**
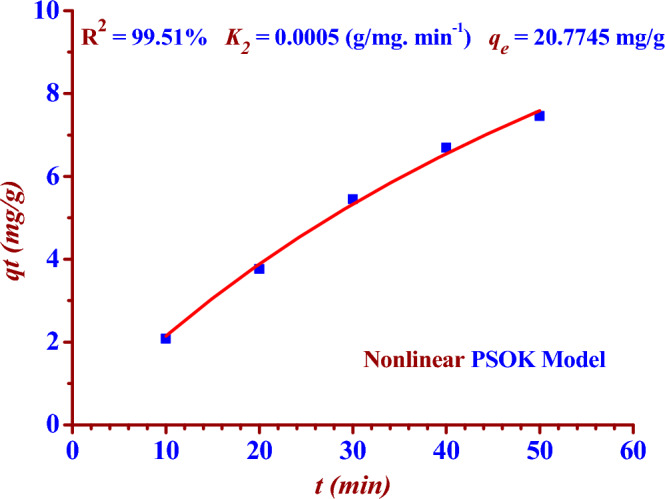
Nonlinear PSOK model for (^127^I^−^) adsorption.

The adsorption results had been recorded in Table 2, were linear fitted to two kinetic models (PFOK model) and (PSOK model) as indicated in the two Figs. 8 and 9. Nonlinear fitting to PFOK and PSOK models ARE shown in the two Figs. 10 and 11. The regression coefficients and adsorption rate constants (*K*_1_ and *K*_2_) were calculated as shown in the Table 3.

**Table 2:** The calculations of log (*q*_*e*_* − q*_*t*_) and (t/*q*_*t*_) at different contact time (t).

t (min)	C_o_ (ppm)	C_t_ (ppm)	Removal (%)	log (q_e_ − q_t_)	$\frac{{\varvec{t}}}{{{\varvec{q}}_{{\varvec{t}}} }}$
10	0.5014	0.3972	20.78	1.934	4.798
20		0.3131	37.55	1.655	5.311
30		0.2289	54.35	1.267	5.505
40		0.1668	66.73	0.836	5.977
50		0.1286	74.35	0.434	6.706
60		0.0514	89.75	1.934	4.798

**Table 3:** Pseudo-first and second-order kinetics parameters for the adsorption of (^127^I^−^) from an aqueous solution (0.5014 ppm) by Nano ZnO/MWCNT.

Kinetic Models	Regression coefficients	Adsorption rate constants	q_e,_	q_e,_ experimental
PFOK (Linear Form)	R^2^ = 99.46%	*K*_1_ =− (Slope * 2.303) *K*_1_ = 0.0880 min^−1^	= Exp (Intercept) = 10.0749 mg/g	9.00 mg/g
PSOK (Linear Form)	R^2^ = 96.42%	*K*_2_ = (Slope)^2^ * (Intercept)^−1^ *K*_2_ = 0.0005 g/mg min	= (Slope)^−1^ = 22.32 mg/g	
PFOK (Nonlinear Form)	R^2^ = 99.61%	*K*_1_ = 0.0188 min^−1^	12.4217 mg/g	
PSOK (Nonlinear Form)	R^2^ = 99.51%	*K*_2_ = 0.0005 g/mg. min	20.7745 mg/g	

### Pseudo-first order kinetic (PFOK) model

The two Eqs. (3) and (4) represent linear and nonlinear pseudo-first -order kinetic models respectively^14,32,33^.

3 $$ \ln \left( {q_{e} - q_{t} } \right) = \ln q_{t} - K_{1} t $$

4 $$ q_{t} = q_{e} \left( {1 - \exp \left( { - K_{1} t} \right)} \right) $$

### Pseudo second order kinetic (PSOK) model

The two Eqs. (5) and (6) represent linear and nonlinear pseudo-first -order kinetic models respectively^14,32,33^.

5 $$ \frac{{\text{t}}}{{{\text{q}}_{{\text{t}}} }} = \frac{1}{{{\text{K}}_{2} {\text{q}}_{{\text{e}}}^{2} }} + \frac{1}{{{\text{q}}_{{\text{e}}} }}{\text{t}} $$

6 $$ {\text{q}}_{{\text{t}}} = \frac{{{\text{q}}_{{\text{e}}}^{2} {\text{K}}_{2} {\text{t}}}}{{{\text{q}}_{{\text{e}}} {\text{K}}_{2} {\text{t}} + 1}} $$

where (*q*_*e*_ and *q*_*t*_) are the adsorption capacities (mg/g) at equilibrium and time (t), respectively. The adsorption rate constants for the pseudo-first order (PFOK) and second-order kinetic (PFOK) models are *K*_*1*_ (min^-1^) and *K*_*2*_ (g/mg.min), respectively.

The results in the Table 3 show the linear PFOK model are a better model fitting the kinetics of the iodine (^127^I^−^) adsorption than that of linear PSOK model; (R^2^_PSOK_ < R^2^_PFOK_)**.** According to linear and nonlinear estimation of PFOK and PFOK models, the experimental qe value is practically similar to that obtained theoretically in the case of PFOK model. The larger rate constant (*K*_*1*_) of the PFOK model as compared to the smaller rate constant (*K*_*2*_) of the PSOK model indicates the adsorption process is fast^28,29^.

## Adsorption isotherm analysis

The adsorption capacity of (Nano ZnO/MWCNTs) for iodide (^127^I^−^) from aqueous solutions was evaluated by using Langmuir, Freundlich and Temkin adsorption isotherm models in order to describe the behaviour of adsorption. Langmuir model attributes to the formation of monolayer adsorption on the outer surface of the adsorbent. The Freundlich isotherm model has suggested multilayer adsorption of target ions onto the surface of adsorbent. Temkin isotherm focuses on the premise that the free energy of sorption is a property of surface coverage. Some papers fit the results of adsorption into linear isotherm models but others fit the results of adsorption into non-linear isotherm models for estimating the isotherm parameters^14,32,33^.

### Langmuir adsorption isotherm model

**Figure 12:**
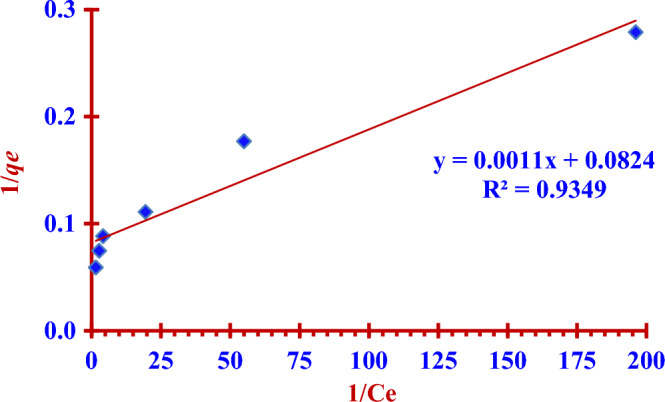
Linear Langmuir isotherm fitting for (^127^I^−^) adsorption by using the (Nano ZnO/MWCNTs).

**Figure 13:**
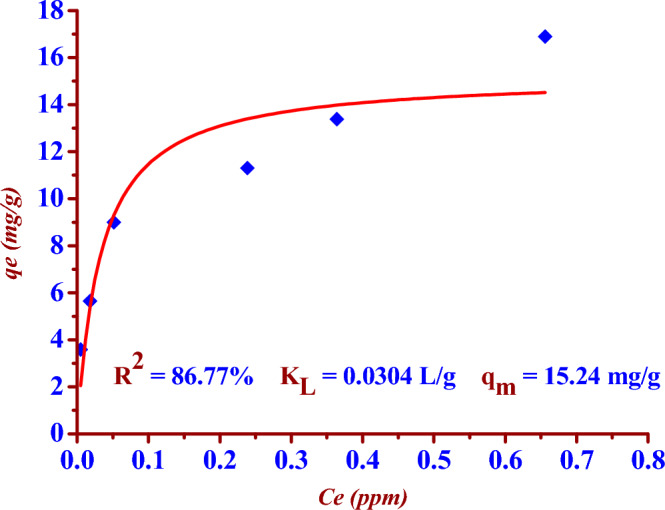
Nonlinear Langmuir isotherm fitting for (^127^I^−^) adsorption by using the nano adsorbent (Nano ZnO/MWCNTs).

The two Eqs. (7) and (8) represent linear and nonlinear Langmuir isotherm formula respectively^14,32,33^.

7 $$ \frac{1}{{{\text{q}}_{{\text{e}}} }} = \frac{1}{{{\text{q}}_{{\text{m}}} }} + \left( {\frac{1}{{{\text{q}}_{{\text{m}}} {\text{K}}_{{\text{L}}} }}} \right)\frac{1}{{{\text{C}}_{{\text{e}}} }} $$

8 $$ {\text{q}}_{{\text{e}}} = \frac{{{\text{q}}_{{\text{m}}} {\text{K}}_{{\text{L}}} {\text{C}}_{{\text{e}}} }}{{\left( {1 + {\text{K}}_{{\text{L}}} {\text{C}}_{{\text{e}}} } \right)}} $$

where *K*_L_ (L/mg) is Langmuir adsorption constant, C_e_ (mg/L) is the iodide ions (^127^I^−^) concentration at equilibrium, *q*_e_ (mg/g) is the adsorption capacity at equilibrium but *q*_max_ (mg/g) is the maximum adsorption capacity (Figs. 12 and 13).

**Figure 14:**
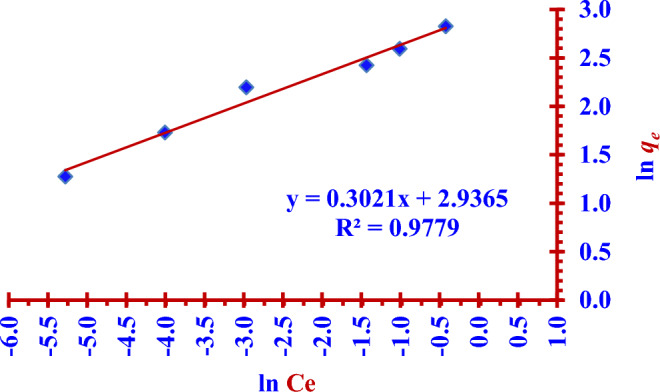
Linear Freundlich isotherm fitting for (^127^I^−^) adsorption by using the (Nano ZnO/MWCNTs).

**Figure 15:**
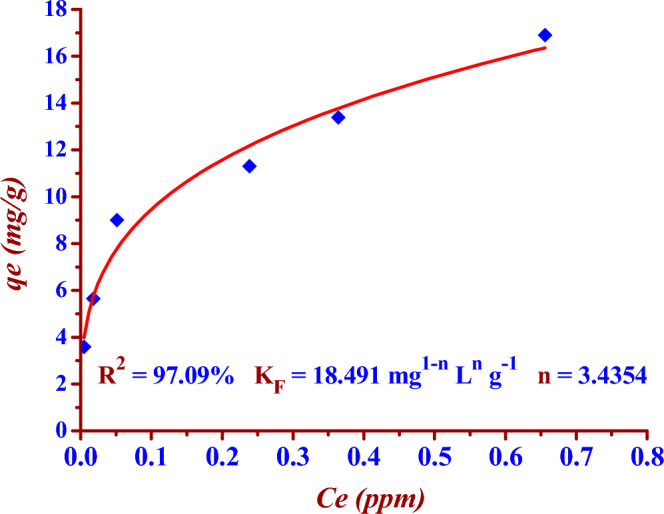
Nonlinear Freundlich isotherm fitting for (^127^I^−^) adsorption by using the nano adsorbent (Nano ZnO/MWCNTs).

The two Figs. 14 and 15 represent the linear and nonlinear fitting to Langmuir isotherm model for the (^127^I^−^) adsorption using the nano adsorbent (Nano ZnO/MWCNTs).

### Freundlich adsorption isotherm model

The two Eqs. (9) and (10) represent nonlinear and linear Freundlich isotherm formula respectively^14,32,33^.

9 $$ {\text{ q}}_{{\text{e}}} = {\text{K}}_{{\text{F}}} {\text{C}}_{{\text{e}}}^{{1/{\text{n}}}} $$

10 $$ {\text{ln q}}_{{\text{e}}} = {\text{ln K}}_{{\text{F}}} + \frac{1}{{\text{n}}}{\text{ln C}}_{{\text{e}}} $$

where C_e_ (mg/L) is the concentration of iodide ions (^127^I^−^) at equilibrium time, *q*_e_ (mg/g) is the adsorption capacity at equilibrium time. *K*_F_ (mg^1−n^ L^n^ g^−1^) is called the Freundlich adsorption constant which refers to the strength of the adsorptive bond. The heterogeneity factor (n) represents the adsorption intensity. In addition, in the case of (1 < *n* < 10), this indicates a favourable adsorption.

(i) In the case of (n < 1), this indicates chemical adsorption.
(ii) In the case of (*n* > 1), this indicates physical adsorption.
(iii) In the case of (*n* = 1), this indicates a partitioning between the chemical and physical adsorption, it is a linear adsorption process.

The two Figs. 14 and 15 represent the linear and nonlinear fitting to Freundlich isotherm model for the (^127^I^−^) adsorption using the nano adsorbent (Nano ZnO/MWCNTs).

### Temkin adsorption isotherm model

**Figure 16:**
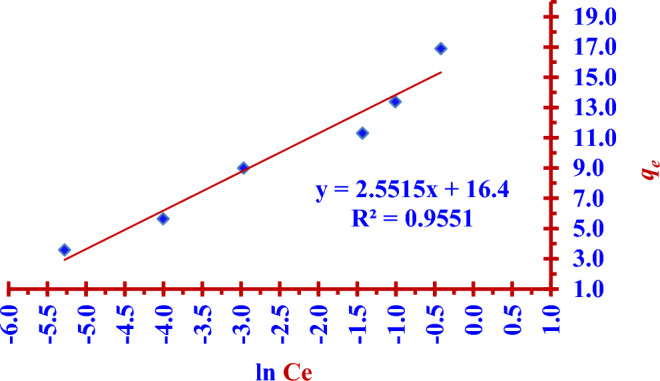
Linear Temkin isotherm fitting for (^127^I^−^) adsorption by using the (Nano ZnO/MWCNTs), at T = 298 K.

**Figure 17:**
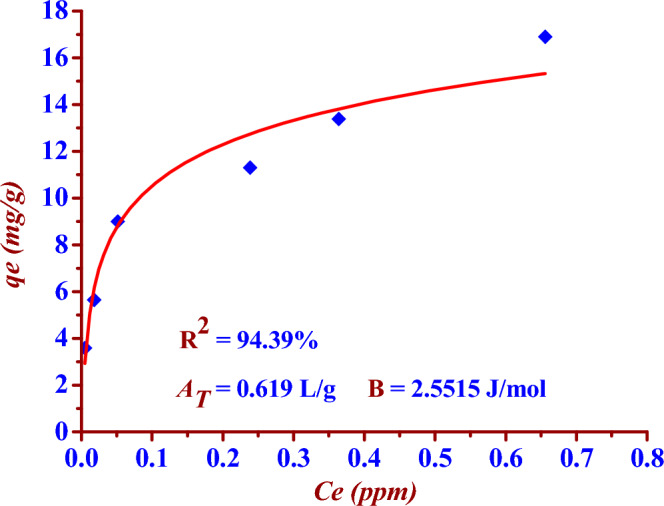
Nonlinear Temkin isotherm fitting for (^127^I^−^) adsorption by using nano adsorbent (Nano ZnO/MWCNTs).

This model is based on assumption that heat of adsorption will not remain constant and decreases due to interaction between the sorbent and the sorbate. The two Eqs. (11) and (12) represent nonlinear and linear Temkin isotherm formula respectively^14,32,33^.

11 $$ {\text{q}}_{{\text{e}}} = \frac{{{\text{RT}}}}{{\text{b}}}{\text{ln }}\left( {{\text{A}}_{{\text{T}}} {\text{C}}_{{\text{e}}} } \right) $$

12 $$ {\text{q}}_{{\text{e}}} = \frac{{{\text{RT}}}}{{{\text{b}}_{{\text{T}}} }}{\text{ln A}}_{{\text{T}}} + \left( {\frac{{{\text{RT}}}}{{\text{b}}}} \right){\text{ln C}}_{{\text{e}}} $$

13 $$ q_{e} = B\ln A_{T} + B\ln C_{e} $$

where C_e_ (mg/L) is the concentration of iodide ions (^127^I^−^) at equilibrium time, *q*_e_ (mg/g) is the adsorption capacity at equilibrium time, *A*_*T*_ (L/g) is Temkin isotherm equilibrium binding energy constant,* b* is Temkin isotherm constant, T (K) is the absolute temperature,* R* is the gas constant of 8.314 (J/mol·K) and B is a constant related to heat of sorption. In case of (B > 0), the model indicates the adsorption process is an exothermic reaction. The two Figs. 16 and 17 represent the linear and nonlinear fitting of Temkin isotherm model for the (^127^I^−^) adsorption by using the nano adsorbent (Nano ZnO/MWCNTs).

In the presented study Freundlich isotherm model is a better model fitting the adsorption iodine (^127^I^−^) on the nano adsorbent (Nano ZnO/MWCNT) in comparison to the Langmuir isotherm model; (R^2^_Langmuir_ < R^2^_Freundlich_). There is an excellent linear fitting with the Freundlich isotherm adsorption model (97% < R^2^_Freundlich_), as shown in the two Figs. 14 and 15. Heterogeneity factor (*n*) = 3.3102 and it is between (1 and 10). This indicates a favourable adsorption of iodide (^127^I^−^) onto the (Nano ZnO/MWCNT) with multilayer physical interaction; (*n* > 1) as shown in the Table 4. The parameters of nonlinear Langmuir isotherm model were evaluated as (*q*_*max*_ = 15.24 mg/g) and (K_L_ = 0.030 L/g). According to Temkin isotherm model the adsorption process is an exothermic reaction; (*B* = 2.5515 > 0).

**Table 4:** Langmuir, Freundlich and Temkin adsorption isotherm models for the adsorption of iodide (^127^I^−^) from aqueous solutions on the nano adsorbent Nano ZnO/MWCNT under optimum conditions.

Adsorption isotherm	Regression coefficients	Parameters
Langmuir model (linear form)	R^2^ = 93.49%	*q*_*max*_ = (Intercept)^−1^ = 12.14 mg/g *K*_*L*_ = Intercept/ Slope = 0.075 L/g
Langmuir model (nonlinear form)	R^2^ = 86.77%	*q*_*max*_ = 15.24 mg/g *K*_*L*_ = 0.030 L/g
Freundlich model (linear form)	R^2^ = 97.79%	*K*_*F*_ = Exp (Intercept) = 18.850 mg^1−n^ L^n^ g^−1^ *n* = (Slope)^−1^ = 3.3102 (*n* > 1); Physical adsorption
Freundlich model (noninear form)	R^2^ = 97.79%	*K*_*F*_ = 18.491 mg^1−n^ L^n^ g^−1^ *n* = 3.4354 (*n* > 1); Physical adsorption
Temkin model (nonlinear and linear form)	R^2^ = 95.51%	B = 2.5515 J/mol *A*_*T*_ = 0.619 L/g

## Removal of radioactive iodine (^131^I^−^) from aqueous solution

**Figure 18:**
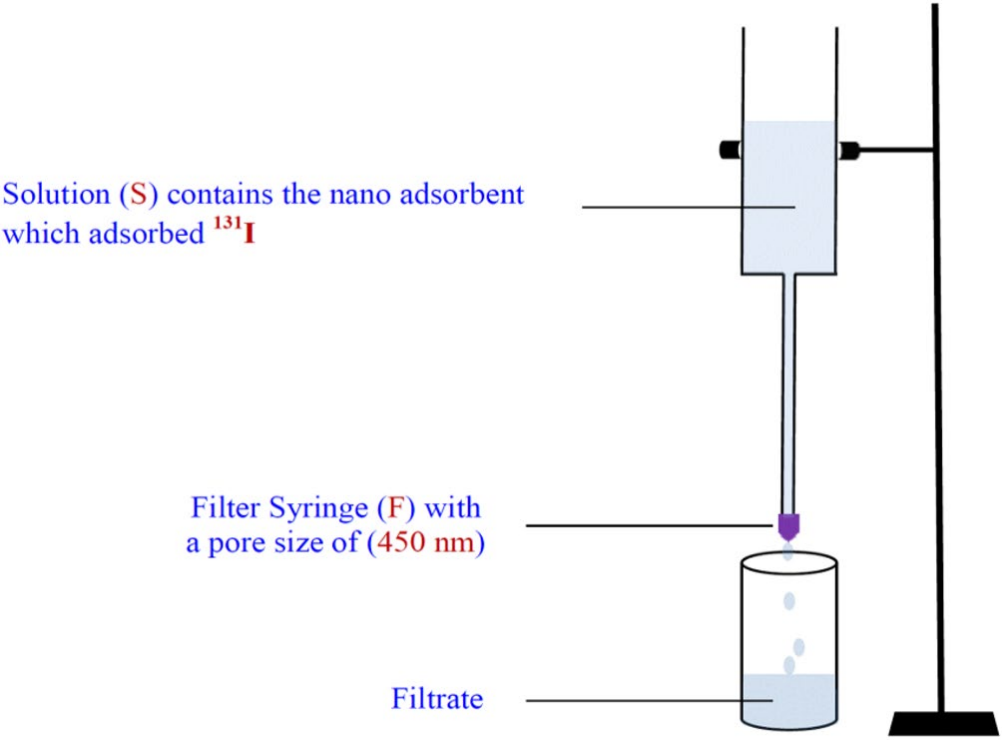
Filtrating system for separating (Nano ZnO/MWCNTs) which adsorbed (^131^I^−^) from the aqueous solution (S).

A solution (200 ml) of (^131^I^−^) was prepared and was divided into two equal volumes each (100 ml) of the same activity (20 µCi). One of them was used as a standard solution (S_d_) to consider the effect of physical decay of radioactive iodine (^31^I^−^) while the nano adsorbent (Nano ZnO/MWCNTs) was applied to the other solution (S). The dose (10 mg) of nano adsorbent was added to the solution (S) in an acidic medium of (pH = 5) with shaking at (200 rpm) for contact time (60 min) at (25 °C) and was allowed to equilibrate for time (t = 2 days). After that, the solution (S) was filtered by a syringe filter (F_S_) with a pore size (450 nm) to separate the used nano adsorbent which had adsorbed (^131^I), as illustrated in Fig. 18. This step represents the first cycle in the removal process.

**Figure 19:**
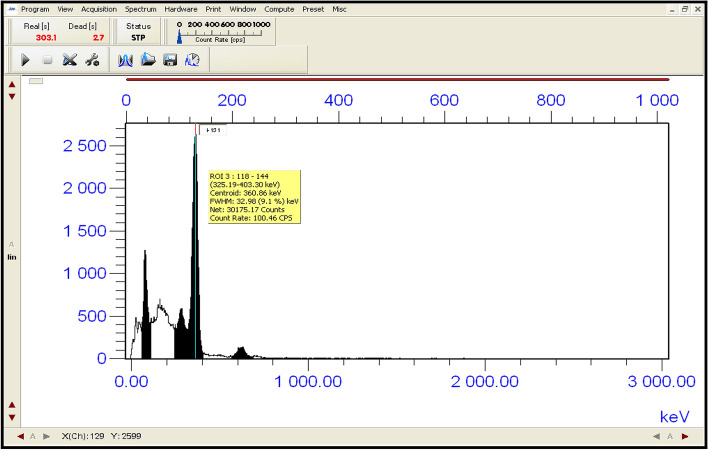
shows the measurement of radioactive iodine (^131^I^−^) in standard solution (S_d_) after 2 days from the preparation.

**Figure 20:**
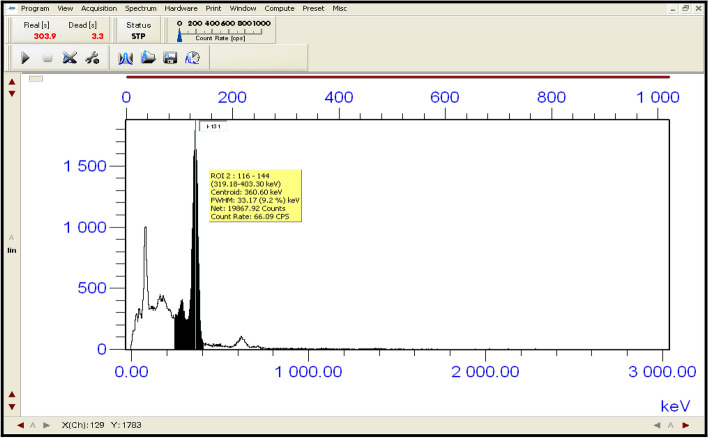
indicates the measurement of radioactive residual iodine (^131^I^−^) in the solution (S) after applying the nano adsorbent and filtration.

**Figure 21:**
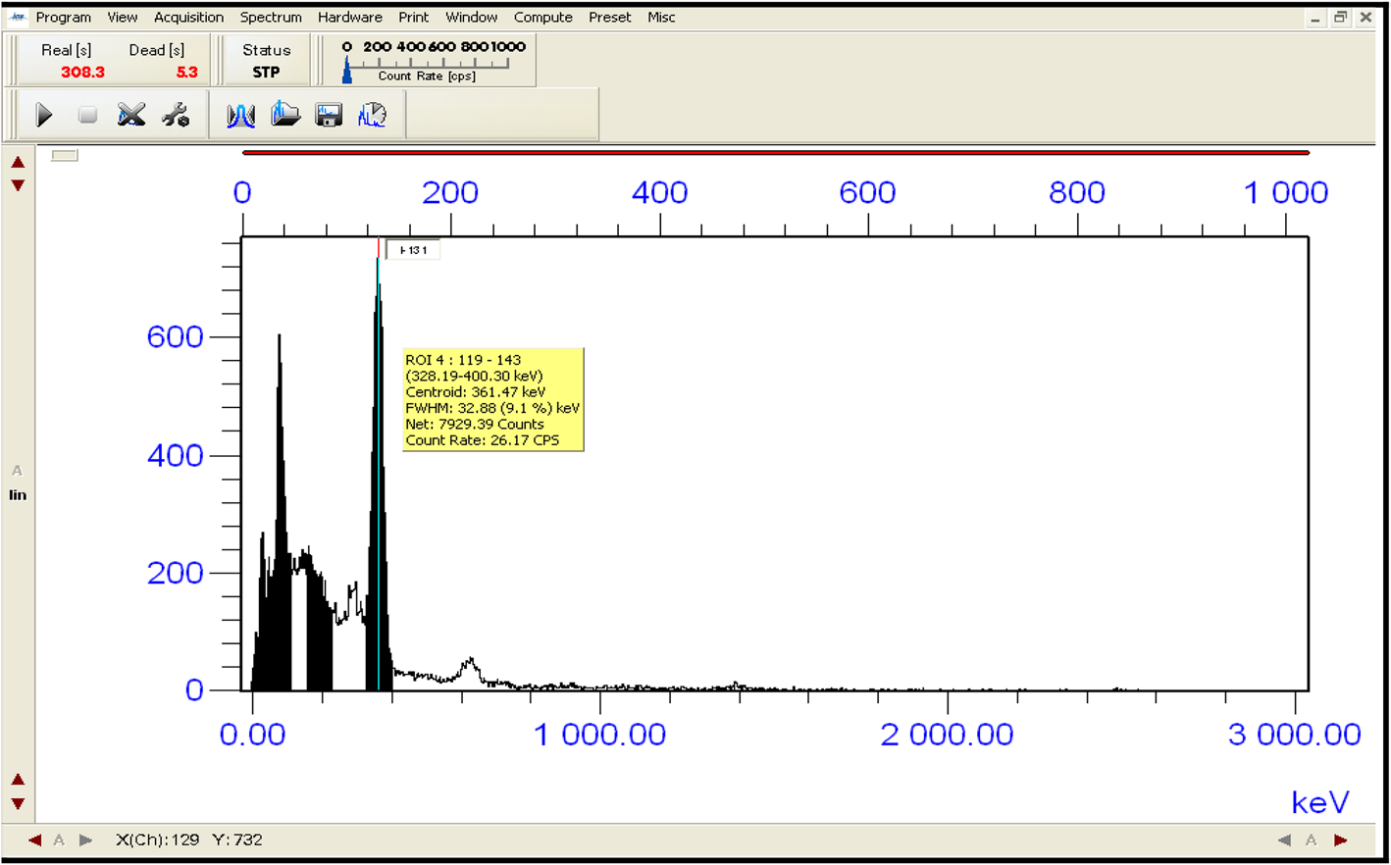
The measurements of the radioactive residual iodine (^131^I^−^) in the syringe filter (F_S_) that is used in the first cycle of the removal process.

Both areas; (Area_o_ and Area_t_) under peaks in the two spectra of standard and filtrate of the same volume, respectively were measured by using the NaI(Tl) scintillation detector to calculate the removal efficiency (%) according to the Eq. (14). Geometry between the NaI (Tl) scintillation detector and the filtrate was at a level to minimize the peaks summing effect and the dead time to be less than (1%). The spectra of standard solution, filtrate and the residual radioactive iodine (^131^I^−^) in the syringe filter (F_S_) are shown in Figs. 19, 20 and 21 respectively. Spectral analysis and the areas under the peaks at energy (360 keV) were calculated by using Genie 2000 software. In the case of the first cycle in the removal process; (Area_o_ = 30,175.17 ± 75.44) while (Area_t_ = 19,867.92 ± 59.61). The removal efficiency of nano adsorbent (Nano ZnO/MWCNTs) for (^131^I) was (34.16%).

14 $$ {\text{Remval Efficiency }}\left( {{\% }} \right) = \frac{{\left( {{\text{Area}}_{{\text{o}}} - {\text{Area}}_{{\text{t}}} } \right)}}{{{\text{Area}}_{{\text{o}}} }} \times 100 $$

**Figure 22:**
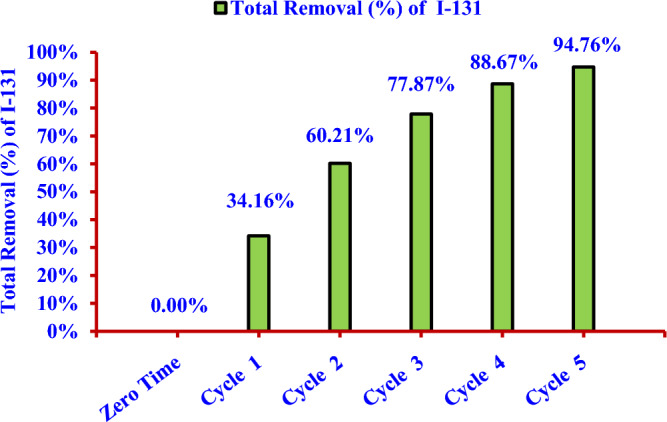
Removal efficiencies for iodine (^131^I^−^) from the aqueous solution by using the nano adsorbent (Nano ZnO/MWCNTs) through 5 cycles.

The filtrate from the first cycle was used to repeat the removal process and was considered as the second cycle and so on until the fifth cycle. The separating time between applying the nano adsorbent and filtration process in each cycle was 2 days to equilibrate. In each cycle, the spectral analysis of filtrate and standard of the same volume were carried out exactly as in the first cycle to calculate the removal efficiency (%). The standard and tackled filtrate from the removal process in each cycle had the same decay time. Finally, the total removal efficiency (%) of nano adsorbent for (^131^I^−^) from the solution (S) was calculated. The removal efficiency (%) for radioactive iodine (^131^I^−^) has been reached (94.76%) after the fifth cycle as indicated in the Fig. 22.

**Figure 23:**
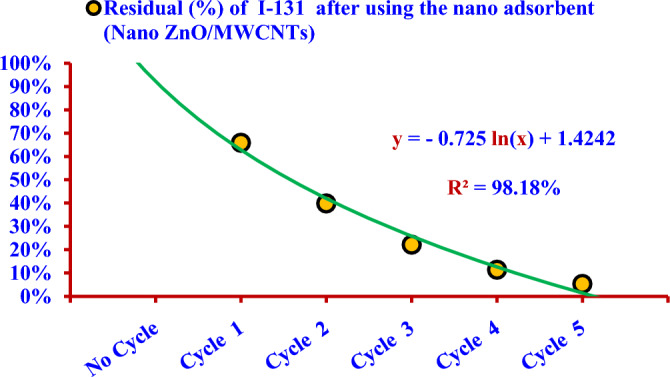
Residual percentage of iodine (^131^I^−^) after using the nano adsorbent (Nano ZnO/MWCNTs) versus the number of treat.

The relation between the residual radioactive iodine (^131^I^−^) and the number of treats is logarithmic, as illustrated in the Fig. 23. The results have attributed to the nano adsorbent dose of (50 mg/100 ml) is the optimum dose for adsorbing (^131^I) in one cycle of treatment.

**Figure 24:**
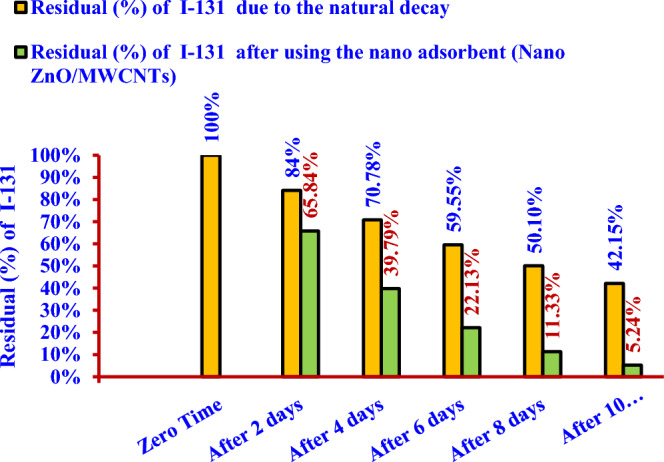
Comparison between the residual iodine (^131^I^−^) from the aqueous solution (S) after using the nano adsorbent (Nano ZnO/MWCNTs) for 10 days and the residual due to the natural decay.

Figure 24 shows the percentage of residual radioactive iodine (^131^I^−^) in the solution (S) after one cycle reached (65.84%) during 2 days, and it was less than that (84%) due to the natural decay. The percentage of residual radioactive iodine (^131^I^−^) in the same solution (S) reached (5.24%) after five cycles of treatment during 10 days; it was less than that (42.15%) due to the natural decay.

The presented technique is suitable for adsorbing all isotopes of radioactive iodine like; (^125^I, ^129^I, and ^131^I) from the aqueous solutions because all iodine isotopes have the same chemical properties^18^. This technique is a simple method with low costs to treat the contaminated water from undesired trace dissolved isotopes of iodine. Besides that, this technique is environmentally friendly. In the case of stable iodine (^127^I) solution (0.05 ppm, 1.0 ppm, 1.5 ppm,…), the probability of collisions between the nano adsorbent particles and the ions of stable iodine (^127^I^−^) is great. On the other hand, for radioactive iodine (^131^I) solution, the probability of collisions between the nano adsorbent particles and the dissolved traces ions of radioactive iodine (^131^I) of activity (A = 20 µCi) is small. Some previous studies illustrated that the adsorbents may have low removal efficiency for (^131^I^−^) from aqueous solutions^34,35^.

On 11 March 2011. Radioactive iodine (^131^I) was detected in raw water in Fukushima and neighboring prefectures. It was removed from the river water sample by using powdered activated carbon (PAC) as an adsorbent with a dose of (25 mg PAC/1L of river water sample). The removal efficiency was about (36%). This percentage was increased from (36%) to (59%), in the case of chlorination before using PAC^34^.

The Nano-composites (Graphene Oxide/Chitosan Sponge) was used to remove stable iodine (^127^I). The optimum dose was (2 mg/50 ml) at (pH = 7.2) during contact time of (24 h) and the removal efficiency was (94.9%). In case of radioactive iodine (^131^I), the dose (4 mg/50 ml) at (pH = 7.2) give removal efficiency (92.6%) after contact time of (24 h)^35^.

## Comparison between the nano adsorbents Nano ZnO/MWCNTs with other nano adsorbents used to remove iodine form water

**Figure 25:**
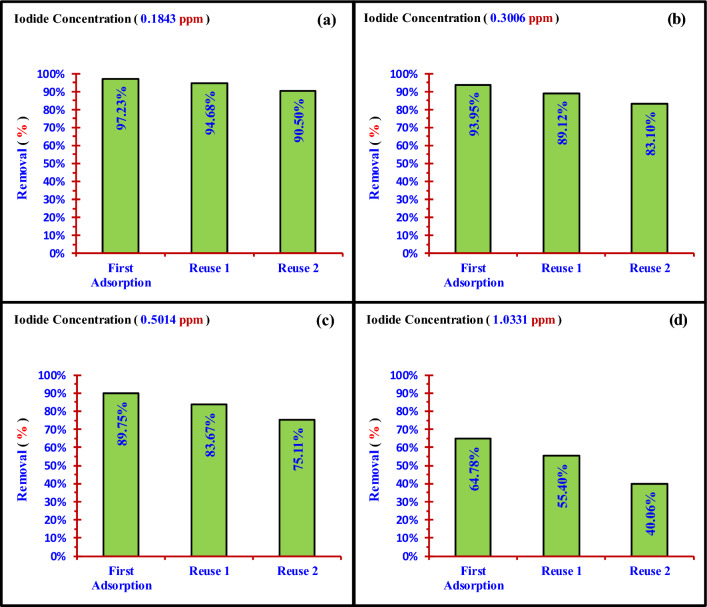
Removal efficiency obtained due to Nano ZnO/MWCNTs reuse at initial concentrations; (**a**) 0.1843 ppm (**b**) 0.3006 ppm (**c**) 0.5014 ppm (**d**) 1.0331 ppm.

The Table 5 indicated the maximum adsorption capacity (15.24 mg/gm) of the prepared nano adsorbent (Nano ZnO/MWCNTs) is less than that of other nano adsorbents. Therefore, this study has introduced an economical nano adsorbent (Nano ZnO/MWCNTs) which is suitable for treating the polluted water from low iodide concentrations. This nano adsorbent can be reused two times for the adsorption of iodide, as shown in the Table 6 and Fig. 25.

**Table 5:** Shows a comparison between different nano adsorbents used to remove iodine from aqueous solutions with the presented nano adsorbent (Nano ZnO/MWCNTs).

Nano adsorbent	Best conditions of adsorption		Maximum adsorption capacity (mg/g)	References
	Contact time	pH		
Mn_3_O_4_ PANI	10 min	5.0	126.1	Yin^15^
Ag Cu-based MOFs	450 min	3.0	247.1	Gong^17^
silver/iron oxide nanocomposites	180 min	7.0	847	Zia^20^
Core–shell ZnO/Cu_2_O encapsulated Ag NPs	–	3.0	217.4	Chen^21^
Graphene Oxide/Chitosan Sponge	24 h	7.2	30.49	Suksompong^35^
Nano ZnO/MWCNTs	60 mim	5.0	15.24	Present work

**Table 6:** Removal efficiency obtained due to the Nano ZnO/MWCNTs reuse for adsorption of (I^−^) at 0.1843 ppm, 0.3006 ppm, 0.5014 ppm and 1.0331 ppm.

C_o_ (ppm)	First Adsorption		Reuse 1		Reuse 2	
	C_t_ (ppm)	Removal (%)	C_t_ (ppm)	Removal (%)	C_t_ (ppm)	Removal (%)
0.1843	0.0051	97.23	0.0098	94.68	0.0175	90.50
0.3006	0.0182	93.95	0.0327	89.12	0.0508	83.10
0.5014	0.0514	89.75	0.0819	83.67	0.1248	75.11
1.0331	0.3639	64.78	0.4608	55.40	0.6192	40.06

## Conclusions

The presented work shows a new study for removing the stable iodine (^127^I) isotope and radioactive iodine isotope (^131^I) from the aqueous solutions. The nanocomposite (Nano ZnO/MWCNTs) which had been synthesized by the method of arc discharge in previous work was used as a nano adsorbent. The optimal conditions for maximum removal of iodide (^127^I^−^) from aqueous solutions according the obtained results were the following; the optimum dose of (Nano ZnO/MWCNTs) was (5.0 mg /100 ml) in acidic medium of (pH = 5) with shacking (200 rpm) in a shaking water bath for contact time (60 min) at constant temperature (25 °C). After filtering the iodide (^127^I^−^) solution to separate the used nano adsorbent, the residual iodide (^127^I^−^) in the filtrated solution was measured at a maximum UV wavelength absorbance at (225 nm)**.**

The removal efficiency (%) of (Nano ZnO/MWCNTs) for iodide (^127^I) from aqueous solutions has reached (97.23%, 89.75%, and 64.78%) at the initial iodide concentrations (0.1843 ppm, 0.5014 ppm and 1.0331 ppm), respectively. In addition, the prepared nano adsorbent (Nano ZnO/MWCNTs) can be reused two times for the adsorption of iodide from aqueous solutions. The PFOK model is a better model fitting the kinetics of the adsorption of iodide (^127^I^−^) from water by using the (Nano ZnO/MWCNTs) than that of the PSOK model.

The maximum adsorption capacity is (15.24 mg/g) according to nonlinear Langmuir isotherm model. The adsorption process is favourable (multilayer physical interactions) according to Freundlich isotherm model since the heterogeneity factor is (3.3102). Temkin isotherm model has indicated the exothermic nature of the adsorption process; (*B* = 2.5515 > 0).

In the case of the radioactive iodine (^131^I^−^) solution (20 µCi), the dose of (10 mg) of (Nano ZnO/MWCNTs) was added to 100 ml of the(^131^I^−^) solution in acidic medium (pH = 5) at constant temperature (25 °C) for contact time (60 min) with shaking (200 rpm) in a shaking water bath and allowed to equilibrate for 48 h. The (^131^I^−^) solution was filtered by using a syringe filter with a pore size (450 nm) in order to separate the used nano adsorbent. The filtrated solution from the first cycle of treatment was used to repeat the removal process and this was considered as the second cycle and so on until the fifth cycle.

The removal efficiency was (34.16%) after the first cycle of treatment and reached (94.76%) after five cycles of treatment, where the percentage of residual radioactive iodine was (5.24%). On the other hand, the percentage of residual radioactive iodine which produced due to the natural decay after 10 days is (42.15%).

## Data Availability

All data generated or analyzed during this study are included in this published article.
